# Wheat and Rice Growth Stages and Fertilization Regimes Alter Soil Bacterial Community Structure, But Not Diversity

**DOI:** 10.3389/fmicb.2016.01207

**Published:** 2016-08-03

**Authors:** Jichen Wang, Chao Xue, Yang Song, Lei Wang, Qiwei Huang, Qirong Shen

**Affiliations:** Jiangsu Provincial Key Lab and Coordinated Research Center for Organic Solid Waste Utilization, Nanjing Agricultural UniversityNanjing, China

**Keywords:** wheat-rice rotation system, bacterial community, growth stage, fertilization regime, dynamic variation

## Abstract

Maintaining soil fertility and the microbial communities that determine fertility is critical to sustainable agricultural strategies, and the use of different organic fertilizer (OF) regimes represents an important practice in attempts to preserve soil quality. However, little is known about the dynamic response of bacterial communities to fertilization regimes across crop growth stages. In this study, we examined microbial community structure and diversity across eight representative growth stages of wheat-rice rotation under four different fertilization treatments: no nitrogen fertilizer (NNF), chemical fertilizer (CF), organic–inorganic mixed fertilizer (OIMF), and OF. Quantitative PCR (QPCR) and high-throughput sequencing of bacterial 16S rRNA gene fragments revealed that growth stage as the best predictor of bacterial community abundance and structure. Additionally, bacterial community compositions differed between wheat and rice rotations. Relative to soils under wheat rotation, soils under rice rotation contained higher relative abundances (RA) of anaerobic and mesophilic microbes and lower RA of aerophilic microbes. With respect to fertilization regime, NNF plots had a higher abundance of nitrogen–fixing *Cyanobacteria*. OIMF had a lower abundance of ammonia-oxidizing *Thaumarchaeota* compared with CF. Application of chemical fertilizers (CF and OIMF treatments) significantly increased the abundance of some generally oligotrophic bacteria such those belonging to the *Acidobacteria*, while more copiotrophic of the phylum *Proteobacteria* increased with OF application. A high correlation coefficient was found when comparing RA of *Acidobacteria* based upon QPCR vs. sequence analysis, yet poor correlations were found for the α- and β- *Proteobacteria*, highlighting the caution required when interpreting these molecular data. In total, crop, fertilization scheme and plant developmental stage all influenced soil microbial community structure, but not total levels of alpha diversity.

## Introduction

Winter wheat (*Triticum aestivum* L.) and summer rice (*Oryza sativa* L.) cultivation rotations, with two crops per year, has been practiced for thousands of years in Asia. Approximately 3.4 Mha is under this system in the Yangtse River Basin in China, representing critical source for food security and livelihood of several hundreds of millions people in the region (Dawe et al., 2004; Timsina and Humphreys, 2006). In addition, the ecosystem of the wheat-rice rotation provides diverse and unique habitats for microorganisms, and in turn, microorganisms influence plant growth both directly through symbiosis and indirectly through nutrient cycling (Ahn et al., 2012).

The wheat-rice rotation is one of the highest yielding production systems (Timsina and Connor, 2001). However, this agricultural ecosystem is now associated with serious environmental and ecological problems due to overly intensive farming, especially excessive CFs inputs (Yang et al., 2016). Examples include the runoff or leaching of nitrate, NH_3_ volatilization, soil acidification (Guo et al., 2010), soil compaction (Hamza and Anderson, 2005), and decrease of microbial biomass and respiration rates (Treseder, 2008; Ramirez et al., 2012). Application of organic materials, as opposed to CFs alone, can improve soil organic matter content, pH and the activity and diversity of soil microbes (Zhong et al., 2010). Soil microbes can be sensitive to environmental stress and play important roles in the nutrient transformation of fertilizers (Paul, 2014; Zhang et al., 2015), and soil-borne microbial communities are known to be impacted by fertilization management scheme (Geisseler and Scow, 2014). Globally, and especially in China, organic or organic–inorganic combined fertilization is partly or greatly replacing the chemical fertilization due to its contribution in maintaining soil fertility (Palm et al., 1997; Mäder et al., 2002). However, how soil bacterial communities respond to different fertilization practices is likely to be complex, especially in the rice-wheat rotation system where soil undergoes alternate aerobic and anaerobic conditions.

Soil bacterial community has been shown to vary with crop and crop growth stage in wheat-rice rotation (Culman et al., 2006). Several factors can influence bacterial communities, including the contrasting water management between wheat and rice cultivation (Wang et al., 2015), and the different root exudates of each crop (Ladygina and Hedlund, 2010; Guong et al., 2012). The effects of fertilization regimes on soil-borne bacterial communities in wheat-rice rotation systems have been investigated before (Ahn et al., 2012; Zhao et al., 2014), however, previous studies have only examined a single growth stage, thereby only providing a snapshot of potential microbial community responses. Therefore, given the dynamics of soil-borne microbial communities in response to crop growth stage, it is essential to track microbial community responses over time to determine the relative impact of fertilizer treatment on soil bacterial communities. To our knowledge, scarce information is available regarding the time course of changes in bacterial communities under different fertilization practices in the wheat-rice rotation ecosystems. We suggest that this information is fundamental to an understanding the sustainability of the system.

In current study, soil samples from a long-term (8 years) field experiment where wheat-rice cultivation fields are given different fertilizer regimes, including no nitrogen fertilizer (NNF), chemical fertilizer (CF), organic–inorganic mixed fertilizer (OIMF), and organic fertilizer (OF). Soil samples were collected throughout eight different stages and subjected to high-throughput sequencing analysis of bacterial community structure and diversity. The main objectives of this study were to: (1) identify which factors mostly strongly impact bacterial communities over time in wheat-rice rotation systems; (2) understand the extent to which impacts of fertilizer treatment are maintained across different stages of the rotation; (3) if the α-diversity varied regularly with different stages and fertilization regimes and (4) if chemical vs. OFs input alter the general balance of copiotrophic (low C use efficiency, fast-growing) versus more oligotrophic taxa (high C use efficiency, slow-growing). We hypothesized that both different fertilization mode and different crop growth stages would impact soil bacterial communities and that microbial taxa with different general life histories would show disparate population dynamics across fertilization regimes and rotation stage.

## Materials and Methods

### Site Description

The long-term experimental field site with the wheat-rice rotation system is situated in Changshu, Jiangsu province, China (31°18′N, 120°37′E, 6 m asl), and was established in 2005. The site has a humid subtropical monsoon climate with an average annual rainfall of ≈1063 mm and mean minimum and maximum temperatures in 2013 of 2 and 36°C, respectively.

Every year, after rice is harvested, wheat is sown using 150 kg seeds ha^-1^ in October or November. Rice is transplanted in June using two seedlings per hill at 13 cm × 28 cm spacing after wheat has been harvested. The rice plots are flooded with 5 cm of standing water from July to September. Crops are harvested manually at ground level by sickle, and the above-ground biomass removed. After the wheat and rice harvests, the plots are plowed to a depth of 20 cm and fertilizers are applied before sowing and planting as basal fertilizer.

### Experimental Design and Soil Sampling

The randomized block plots were 6 m × 7 m in size. Four treatments with three replicates each were established in a randomized block design: (1) NNF, in which 750 kg ha^-1^ superphosphate (12% P_2_O_5_) and 183 kg ha^-1^ muriate of potash (60% K_2_O) were applied; (2) NPK CF, in which 391 kg ha^-1^ urea (46% N), 750 kg ha^-1^ superphosphate and 183 kg ha^-1^ muriate of potash (mixed and pilled) were applied; (3) OIMF, in which 1500 kg ha^-1^ OIMF (11.0% organic C, 12.0% total N, 4.1% P_2_O_5_ and 4.1% K_2_O, mixed and pilled) was applied, with an extra application of 28.5 kg ha^-1^ superphosphate and 48.5 kg ha^-1^ muriate of potash to reach total quantities of N, P_2_O_5_, and K_2_O that were equal to the CF plots; (4) OF, in which 4500 kg ha^-1^ OF only (26.4% organic C, 2.5% total N, 1.6% P_2_O_5_, and 1.3% K_2_O, from composted pig manure and rice straw by Tianniang Ltd., of Changshu, China) was applied. All the fertilizers were applied as basal fertilizers in October 25th, 2012 and June 5th, 2013 after the harvest of rice and wheat, respectively, and before sowing or transplanting of the subsequent crop. The details on the experimental design can be found in Wang et al. (2016).

Bulk soil samples at a depth of 0–20 cm were collected in 2013 in March 1st (Mar, wheat tillering), April 1st (Apr, wheat jointing), May 1st (May, wheat heading), June 4th (Jun, wheat ripening), July 7th (Jul, rice tillering), August 16th (Aug, rice jointing), September 15th (Sep, rice heading), and October 31st (Oct, rice ripening). For each plot, four cores (5 cm in diameter) were collected, which were pooled and sieved (2 mm) to remove the aboveground plant materials, roots and stones. Fresh soil was used for DNA extraction immediately after collection. Sub-samples were frozen at -80°C.

### Soil Properties Analysis

Soil pH and electrical conductivity (EC) were determined with a soil-to-water ratio of 1:2.5. Soil organic carbon (SOC) was determined by the K_2_Cr_2_O_7_ oxidation-reduction titration method (Schollenberger, 1931). The Kjeldahl method was used for total nitrogen (TN) estimation (Page, 1982). Soil available nitrogen (AN, nitrate plus ammonium) was extracted with 2 M KCl and quantified using a BRAN+LUEBBE AutoAnalyzer 3. Soil available phosphorus (AP) was extracted with sodium bicarbonate and then measured by the molybdenum-blue method (Olsen, 1954). Soil available potassium (AK) was extracted with ammonium acetate and measured using a flame atomic absorption spectrophotometer (Brown, 1998). Soil moisture content was determined by drying the soil at 105°C for 12 h. Soil temperature (1 dm) was determined at 14:00 on each sampling day.

### DNA Extraction

DNA was extracted from 0.25 fresh soil with the MoBio PowerSoil^TM^ DNA Isolation Kits (Mo Bio Laboratories, Carlsbad, CA, USA) according to the manufacturer’s instructions. The quantity and quality of DNA extracts were assayed using a Nanodrop ND-2000 UV–VIS Spectrophotometer (NanoDrop Technologies, Wilmington, DE, USA), and the DNA was stored at -80°C for future analyses.

### Bacterial 16S rRNA Gene Amplification and High-Throughput Sequencing

Amplification of 16S rRNA gene V4 hypervariable region was performed using PCR mixers containing 15 μL of Master Mix (Qiagen Inc., Valencia, CA, USA), 0.5 μM 515F primer (5′-GTGCCAGCMGCCGCGGTAA-3′), 0.5 μM 806R primer (5′-GGACTACHVGGGTWTCTAAT-3′; Caporaso et al., 2012), 10 ng DNA template, and ddH_2_O to a final volume of 30 μL. The Illumina sequencing adapter and the reverse primer contain a 6-bp barcode specific for each example. PCR was performed in triplicate for each sample using the following program: 1 min at 98°C for initial denaturing, followed by 35 cycles of 98°C for 10 s, 50°C for 30 s and 72°C for 30 s with the final extension for 5 min at 72°C. After amplification, the triplicate PCRs were pooled and purified using the PCR cleanup Kit (Axygen Biosciences, Union City, CA, USA). Sequencing was performed using the Illumina MiSeq platform (Caporaso et al., 2012) at Novogene Bioinformatics Technology Co., Ltd., Beijing, China.

### Bioinformatics and Statistical Analysis

The UPARSE standard pipeline was applied to analyze the sequence data (Edgar, 2013). Briefly, short reads (<250 bp) sequences were filtered out before downstream analysis. Sequences with ≥ 97% similarity were clustered into operational taxonomic units (OTUs). All sequences were assigned using the RDP classifier to identify taxonomies with a confidence threshold of 0.8 (Wang et al., 2007). An average of 1.9% of the sequences was identified as being of archaeal origin and these sequences were also included in further data analyses.

As the number of sequences per sample ranged from 4,241 to 32,286, the data from each sample was resampled to a depth of 4,241 sequences for each sample using MOTHUR (Schloss et al., 2009), and the resulting new OTU table used for the further analyses. MOTHUR was then used to calculate α-diversity, expressed as Shannon and Simpson indexes, for each sample. Multivariate ANOVAs based on the Shannon and Simpson indexes and 16S rRNA gene copies were performed to determine the effects of stage and fertilization regime on the bacterial diversities and abundance using SPSS 18.0. Hierarchical cluster analysis was carried out based on the Bray–Curtis distances of OTU composition using MOTHUR. Non-metric multidimensional scaling (NMDS), based on the Bray–Curtis distances of phyla relative abundance (RA). To compare the effects of stage, fertilization regime and their interactions on bacterial community structures, permutational multivariate analysis was performed based on OTU RA. Correlations between the Euclidean distance of soil properties and bacterial OTUs RA were calculated using the Mantel test. Redundancy analysis (RDA) was performed to analyze relationships between soil properties and bacterial dominant taxa, and followed by 999 permutations to test significance. All these analyses were performed using the ‘vegan’ package in R (R Core Team, 2014). One-way ANOVAs of soil properties were performed to compare the differences among the four fertilization regimes within the same sampling stage, followed by Tukey’s HSD as a *post hoc* test using SPSS 18.0. Pearson correlation analyses were performed using SPSS 18.0. Linear discriminant analysis effect size (LEfSe) was performed to identify significant differences in bacterial taxa between groups. The Kruskal–Wallis (KW) sum-rank test was used in LEfSe analysis to detect the features with significantly different abundances between assigned classes, and then liner discriminant analysis (LDA) was performed to estimate the effect size of each feature (Segata et al., 2011).

### Quantitative Real-Time PCR Assays

The abundance of total bacterial 16S rRNA gene copies was quantified by quantitative PCR as well the abundances of *Alphaproteobacteria, Betaproteobacteria*, and *Acidobacteria* to complement the results of the high-throughput sequencing. Quantitative PCR (QPCR) assays were performed using the SYBR^®^ Premix Ex TaqTM (Perfect Real Time) kit (TaKaRa Biotechnology Co., Dalian, China) using the 7500 Fast Real-Time PCR System (Applied Biosystems, Branchburg, New Jersey, USA). Each reaction was performed in a 25 μL volume containing 12.5 μL of SYBR^®^ Premix Ex TaqTM (2×, Takara), 0.5 μL ROX Reference dye II (50×, TaKaRa Biotechnology Co., Dalian, China), 10 μL dd H_2_O, 1 μL (∼10–30 ng) DNA template and 0.5 μL (5 μM) of each primer. Bacterial 16S rRNA primers were Eub338 (5′-ACT CCT ACG GGA GGC AGC AG-3′) and Eub518 (5′-ATT ACC GCG GCT GCT GG-3′), *Alphaproteobacteria* class primers were Eub338 (5′-ACT CCT ACG GGA GGC AGC AG-3′) and Alfa685 (5′-TCT ACG RAT TTC ACC YC TAC-3′); *Betaproteobacteria* class primers were Eub338 and Beta510 (5′-TCA CTG CTA CAC GYG-3′); *Acidobacteria* primers were Acid31 (5′-GAT CCT GGC TCA GAA TC-3′); and Eub518 (5′-ATT ACC GCG GCT GCT GG-3′) (Fierer et al., 2005). The PCR protocols for *Alphaproteobacteria, Betaproteobacteria*, and *Acidobacteria* were initiated with an activation step at 95°C for 30 s, followed by 40 cycles with 5 s at 95°C for denaturation and 34 s at 60°C for annealing and extension. Reactions were tested for possible PCR inhibitors contained in DNA extractions by comparing results across serial dilutions. No significant inhibitory effects of DNA extracts was detected. Standard curves were developed by serially diluting plasmids with known positive inserts to final concentrations of 10^8^ to 10^2^ gene copies μL^-1^. QPCR efficiencies were 111, 115, 95, and 98% for bacteria, *Alphaproteobacteria, Betaproteobacteria*, and *Acidobacteria* reactions, respectively, and *R*^2^ values for all four assays were >0.99.

### Sequence Accession Numbers

All sequence data has been deposited in the NCBI Sequence Read Archive (SRA) database under the accession number SRX1117202.

## Results

### Soil Properties

Soil organic carbon content was significantly higher in the OF treatment as compared to the other three fertilizer treatments (**Supplementary Table S1**). OF plots also tended to have higher soil TN content, pH and EC values relative to the other three treatments. However, the CF and OIMF treatments appeared to decrease the soil pH and EC values and increase soil NO_3_^-^ content. Soil NH_4_^+^ content tended to be higher in the CF and OIMF treatments. Soil AK contents were significantly higher for the NNF treatment compared to the other three treatments; however, CF treatment had the lowest AK contents. Soil AP content tended to be higher in OF treatment in August, September, and October.

### Sequence Data and Bacterial Community Composition

Across the total dataset, 1,330,350 sequences from 96 soil samples were grouped into 13,379 OTUs at the 97% similarity cut-off level. An average of 98.1% of the sequences were classified as belonging to the bacteria, while 1.9% of them were designated as being of archaea. The most dominant phylum across all samples was the *Proteobacteria*, which accounted for an average of 34.0% of the total sequences, followed by *Acidobacteria, Chloroflexi*, and *Actinobacteria*, which represented 8.8, 7.3, and 5.9% of sequences, respectively (**Supplementary Table S2**).

### Quantitative PCR of Bacterial 16S rRNA Genes

**FIGURE 1:**
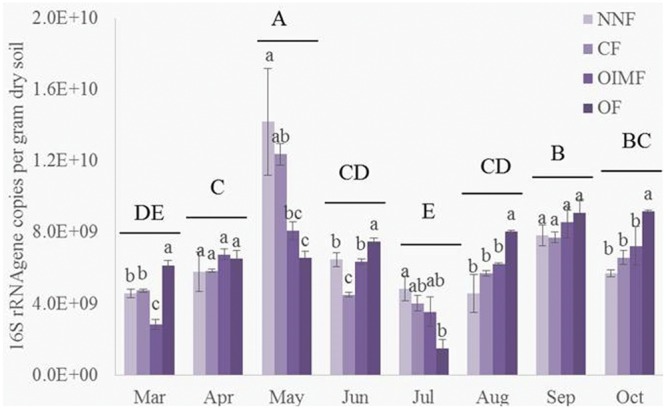
**Abundance of bacterial 16S rRNA genes during eight growth stages of wheat (March–June) and rice (July–October) rotations.** Tukey’s *post hoc* tests were used, and the different letters indicate significant differences (*P* < 0.05) among fertilizer treatments (lowercase) and sampling times (uppercase). Values are presented as mean ± SE (*n* = 3). NNF, no nitrogen fertilizer; CF, chemical fertilizer; OIMF, organic-inorganic mixed fertilizer; OF, organic fertilizer. Data of 16S rRNA gene copies from March to June were collected form Wang et al. (2016).

As shown in the **Table 1**, plant growth stage significantly affected bacterial abundance, which generally increased from 4.56 × 10^9^ g^-1^ dry soil in March to peak level of 1.03 × 10^10^ g^-1^ dry soil in May (**Figure 1**). There after bacterial 16S rRNA gene copies dropped back to a minimum 3.70 × 10^9^ g^-1^ dry soil on the first sampling stage of the rice rotation (July), after which, bacterial abundance again increased and became stable. Fertilization regime had a relative weak influence on the bacterial abundance across the eight stages (**Table 1**), but the effect of the interaction of stage and fertilization was significant.

**Table 1:** Effects of sample stage, fertilizer regime and their interaction on bacterial community abundance, structure and diversity.

	16S rRNA gene copies^a^		Community structures^b^				Community diversity			
	*F*-value	*P*-value	Phylum level		Otu level		Shannon index		Simpson index	
			*R*^2^	Pr (>F)	R^2^	Pr (>F)	*F*-value	*P*-value	*F*-value	*P*-value
Fert	2.146	0.103	0.038	0.003^∗∗^	0.013	0.028^∗^	0.396	0.756	1.662	0.135
Stage	30.956	0.001^∗∗^	0.284	0.001^∗∗^	0.048	0.001^∗∗^	0.601	0.616	1.063	0.398
Fert^∗^Stage	5.407	0.001^∗∗^	0.000	0.993	0.010	0.369	1.104	0.368	1.046	0.426

*^a^Impacts on 16S rRNA gene copy numbers and community diversity were determined by two-way ANOVA.*

*^b^*R*^2^ value (effect size) shows the percentage of variation explained by the category by Permutational multivariate analysis. ^∗^*P*< 0.05, ^∗∗^*P*< 0.01.*

### Effects of Sampling Stage on Bacterial Communities

**FIGURE 2:**
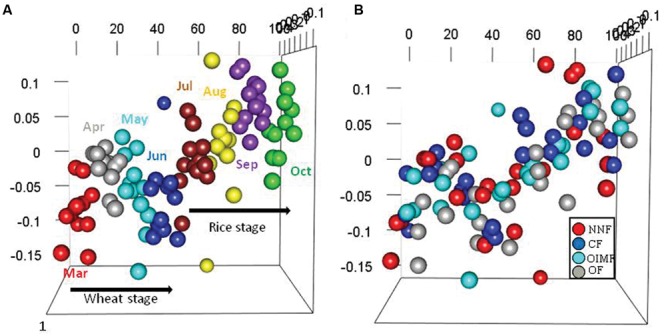
**Bacterial structures under the four fertilization treatments at eight sampling stages in the wheat-rice rotation as analyzed by non-metric multidimensional scaling (NMDS) analysis based on phylum-level abundances.** Plots colored with eight sampling stages **(A)** and four fertilization regimes **(B)** are displayed, respectively. The stress was 0.198, the non-metric fit *R*^2^ value was 0.961 and the linear fit *R*^2^ value was 0.897.

Permutational multivariate analyses based on bacterial RA of phyla and OTUs revealed that stage and fertilization regime both had significant effects on the bacterial community structures (**Table 1**). The plant growth stage yielded higher *R*^2^ values than fertilization regime at both the phylum and OTU levels, indicating that the influence of crops growth stage on the bacterial structure was stronger compared to fertilization regime. NMDS analysis based on the RA of phyla showed that samples of bacterial community structure were grouped by their sampling stages, but not by different fertilization regime, and that adjacent stages had more similar bacterial community structures than more temporally separated stages (**Figure 2**). Similar results were also observed by the cluster analysis based on OTU data (**Supplementary Figure S2**).

**FIGURE 3:**
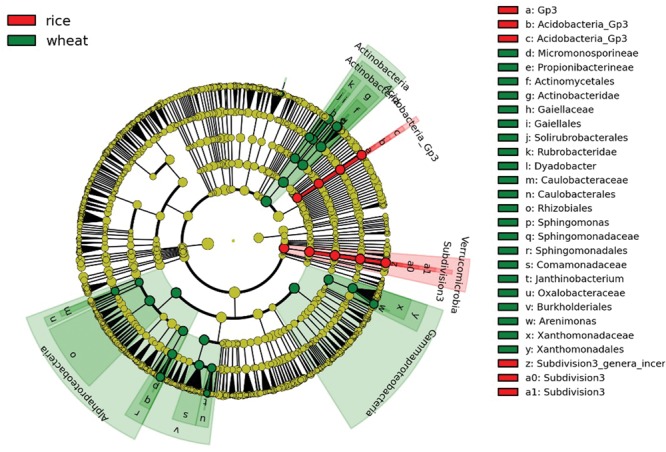
**Cladogram indicating the phylogenetic distribution of bacterial lineages under wheat and rice rotations; lineages with LDA values higher than 2.5 are displayed.** Differences are represented in the color of the most abundant taxa (red indicate rice rotation, green indicate wheat rotation, and yellow are non-significant). Each circle’s diameter is proportional to the given taxon’s relative abundance. Circles represent phylogenetic levels from domain to genus from the inside outwards.

Bacterial community structures were apparently changed varied somewhat during the course of the year as environmental conditions and plant growth stages changed, but the dominant taxa remained *Proteobacteria, Acidobacteria, Chloroflexi*, and *Actinobacteria* (**Supplementary Table S2**). We further divided the eight sampling stages into two groups, representing the wheat rotation (March–June) and the rice rotation (July–October), and performed LEfSe analyses to examine which taxa differed most in RA between the two rotations. The cladogram (**Figure 3**) shows that many taxa were common between the wheat and rice stages of the rotation (shown in yellow), but there were also some specific differences. For example, the RA of *Actinobacteria, Gammaproteobacteria*, and *Alphaproteobacteria* in soils under wheat rotation were higher than in soils under rice rotation, while RA of *Verrucomicrobia* was significantly higher in the rice rotation soils (**Figure 3**; **Supplementary Table S2**).

With respect to bacterial diversity, multivariate ANOVA revealed that plant growth stage and fertilization regime had no significant effects on either Shannon or Simpson indexes (**Table 1**), indicating that bacterial diversity levels were stable both across the plant growth stages and under different fertilization regimes.

### Effects of Fertilizer Treatments on Bacterial Communities

**FIGURE 4:**
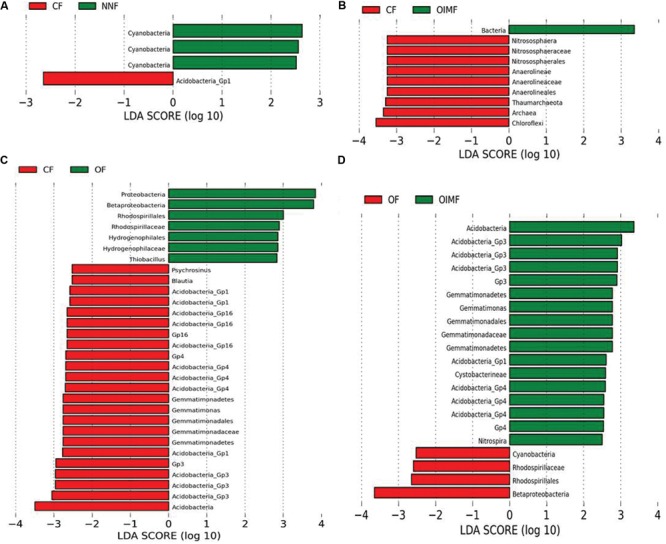
**Liner discriminant analysis coupled with effect size measurements identifies the differentially abundant taxa between fertilizer regimes: NNF vs. CF treatments **(A)**, CF vs. OIMF treatments **(B)**, CF vs. OF treatments **(C),** and OF vs. OIMF treatments **(D)**.** Lineages with LDA values higher than 2.5 are displayed.

Although showing small effect than crop growth stage, fertilization did significantly affect bacterial community structures, as revealed by permutational multivariate analyses. Persistent differences in bacterial taxa patterns between fertilization regimes, regardless of the different growth stages, were revealed by LEfSe analyses (**Figure 4**).

The CF and NNF treatments were compared pairwise to identify the influence of the nitrogen application on the bacterial community compositions (**Figure 4A**). The results revealed that across all stages of the wheat-rice rotation, the NNF treatment resulted in significantly higher (*P*< 0.05) RAs of the *Cyanobacteria* phylum, *Cyanobacteria* class and *Cyanobacteria* order, while the CF treatment had a higher RA of *Acidobacteria*_*GP1*.

The CF and OIMF treatments were compared pairwise to determine how bacterial composition was affected by the partial substitution of OF for CF (**Figure 4B**). The results showed that the RA of Archaea was significantly higher in the CF plots across the whole wheat-rice rotation seasons. Within the Archaea, the phyla *Chloroflexi* and *Thaumarchaeota*, and the classes *Anaerolineae* and *Nitrososphaerales* were also significantly higher in the CF treatment.

Chemical fertilizer and OF treatments were compared pairwise to evaluate the difference in bacterial communities when the soil nutrition was switched from CF to OF alone (**Figure 4C**). The OF treatment exhibited a higher RA of *Proteobacteria* (3.5% higher than CF, **Supplementary Table S2**) and especially the sub-class *Betaproteobacteria*. Input of CFs increased the RA of the *Gemmatimonadetes* phylum, the *Gemmatimonadetes* class and *Gemmatimonadales* order, as well as the *Acidobacteria* phylum (7.6% higher than OF, **Supplementary Table S2**).

The OF and OIMF treatments were compared pairwise to detect the difference in bacterial communities when the nutrition from pure organic matter was partially substituted with CF (**Figure 4D**). Similar to the CF treatment, OIMF also enhanced the RA of the *Gemmatimonadetes* phylum, the class *Gemmatimonadetes* and the order *Gemmatimonadales*, and the RA of the *Acidobacteria* phylum. The application of pure OF increased the RA of *Betaproteobacteria*.

**FIGURE 5:**
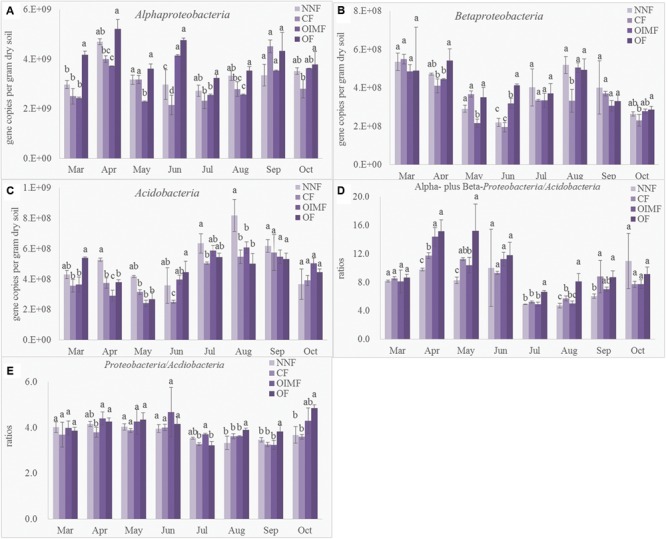
**Abundance of *Alphaproteobacteria***(A)**, *Betaproteobacteria***(B)**, *Acidobacteria***(C)**, ratios between *Alphaproteobacteria* plus *Betaproteobacteria* to *Acidobacteria* deteremined by QPCR **(D)** and ratios between *Proteobacteria* to *Acidobacteria* deteremined by high-thoughput squencing **(E)**.** Values are presented as mean ± SD (*n* = 3). Tukey’s *post hoc* tests were used to compare the effects of fertilizations within every sampling time, and the different letters indicate significant differences among fertilizer treatments (*P* < 0.05).

Quantitative PCR was performed to determine the abundance of the *Acidobacteria* phylum and the α and β sub-classes of the *Proteobacteria* affording comparison with results obtained by high-throughput sequencing (**Figure 5**). Compared with the CF and OIMF treatments, the OF treatment had more abundance of *Betaproteobacteria* (**Figure 5B**), which was in agreemtent with the LEfSe results. *Acidobacteria* tended to be higher in the NNF treatment relative to the other three treatments, but consistantly higher *Acidobacteria* levels were not found in CF and OIMF treatments compared with the OF treatments by QPCR analysis (**Figure 5C**), which was inconsistent with the results from the LEfSe analysis. *Proteobacteria*/*Acidobacteria* (P/A) ratio is suggested be indicative of higher soil nutrient availability, since the specific primer for the phylum *Proteobacteria* could not be confirmed, only α- and β- *Proteobacteria* were determined by QPCR to caculate the P/A ratio. Results showed that the OF treatment tended to have higher P/A ratios than CF and OIMF treatments across all the eight growth stages (**Figure 5D**). This was consistent with the high throughput sequencing that indicated that the OF treatment had the highest P/A ratio (4.05), while the CF treatment had the lowest ratio (3.64; **Figure 5E**). Although the RAs of *Acdiobacteria* by QPCR and by high throughput sequencing were significant (*P*< 0.001, Pearson correlation analysis), the RAs of α- and β- *Proteobacteria* by these two methods were not well correlated (*P*> 0.05).

### Linking Soil Properties with Bacterial Communities

**FIGURE 6:**
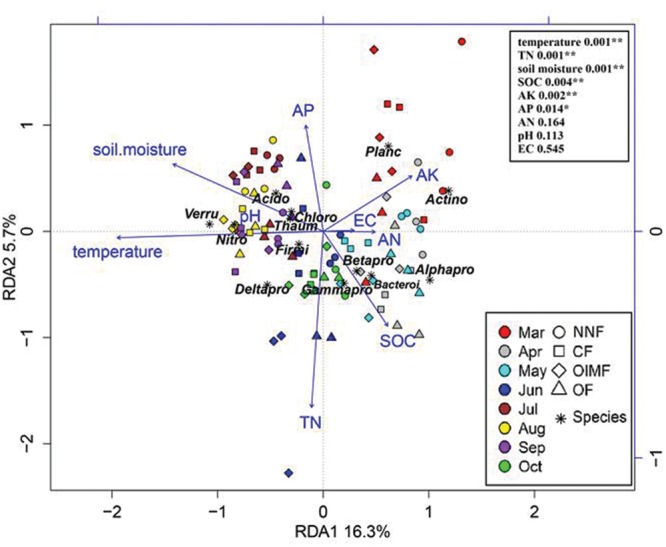
**Redudancy analysis of soil properties and dominant bacterial phyla (>1%, *Proteobacteria* was divided into four subclasses).** Soil property abbreviations: SOC, soil organic carbon; TN, total nitrogen; EC, electrical conductivity; AK, available K; AP, available P; AN, available nitrogen (ammonium and nitrate). Phylum abbreviations: Acido, *Acidobacteria*; Actino, *Actinobacteria*; Thaum, *Thaumarchaeota*; Bacteroi, *Bacteroidetes*; Chloro, *Chloroflexi*; Firmi, *Firmicutes*; Nitro, *Nitrospirae*; Planc, *Planctomycetes*; Verru, *Verrucomicrobia*; Alphapro, *Alphaproteobacteria*; Betapro, *Betaproteobacteria*; Deltapro, *Deltaproteobacteria*; Gammapro, *Gammaproteobacteria*. In the top-right, the soil properties were fitted to the ordination plots using a 999 permutations test (*P*-values).

Redundancy analysis analysis revealed that variation in bacterial community composition was significantly correlated with specific soil properties (**Figure 6**; for additonal detail see **Supplementary Table S3**). For instance, *Betaproteobacteria* and *Bacteroidetes* were positively correlated with soil SOC content and negatively correlated with soil moisture, while *Acidobacteria, Thaumarchaeota*, and *Chloroflexi* tended to prefer high moisture and low SOC content soil conditions. Additionally, as revealed by the RDA biplot and the further permutations test, soil temperature and TN content was the key predictors of bacterial community structure, and soil moisture, SOC, AK, and AP contents also played significant roles. Mantel tests also demonstrated that soil TN content was the factor having greatest influence on bacterial community structure in the wheat rotation, while soil temperature was dominant in the rice rotation (**Table 2**).

**Table 2:** Mantel test (*P*) of bacterial community structure (OTU level) as a function of soil properties.

	Wheat rotation^a^	Rice rotation
SOC^b^	0.841	0.049
Soil moisture	0.683	0.667
Ammonium	0.118	0.213
Nitrate	0.889	0.162
AK	0.146	0.118
AP	0.02	0.803
TN	**0.003**	0.339
EC	0.138	0.435
pH	0.887	0.674
Temperature	0.068	**0.003**

*^a^Significant differences (*P*< 0.01) are indicated in bold.*

*^b^Abbreviations: SOC, soil organic carbon; TN, total nitrogen; EC, electrical conductivity; AK, available K; and AP, available P.*

**FIGURE 7:**
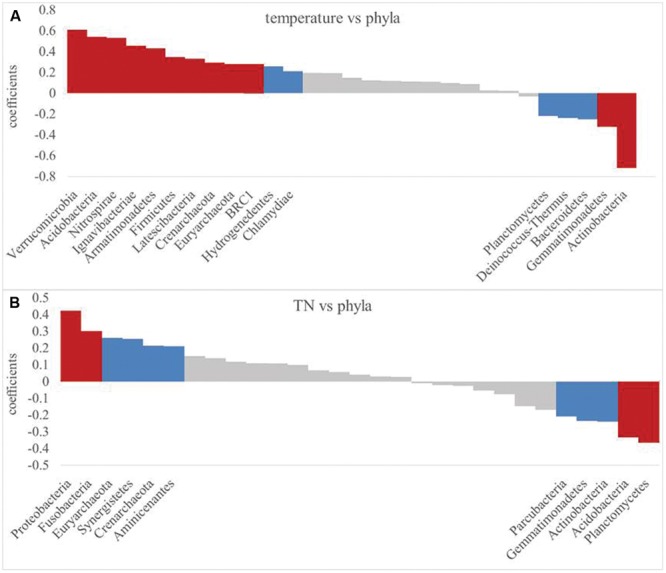
**Pearson relationship between phylum relative abundance and soil temperature **(A)** and TN **(B)**.** Significant difference are indicated by red (*P*< 0.01) and blue (*P*< 0.05).

Since soil temperature and TN contents were the strongest drivers of bacterial community variation. These two soil properties were further correlated with the RA of each phylum as examined by correlation models (**Figure 7**). Most of the bacterial RAs were positively correlated with soil temperature (*P*< 0.01), including *Verrucomicrobia, Acidobacteria*, and seven other phyla, while *Actinobacteria* and *Gemmatimonadetes* were negatively correlated with soil temperature (**Figure 7A**). Soil TN was positively correlated (*P*< 0.01) with the RAs of *Proteobacteria* and *Fusobacteria*, while TN contents and the RAs of *Acidobacteria* and *Planctomycetes* were negatively correlated (**Figure 7B**).

## Discussion

### Effects of Sampling Stage on Bacterial Communities

In present study, plant growth stage was determined to be the most important factor for explaining the observed variation in soil bacterial community structure. Interestingly, bacterial diversity remained steady over the different stages, which did not support our hypothesis mentioned before. Thus, although bacterial community composition fluctuated over time, changes in RA and species replacements were driving these differences as opposed to species loss or gain.

It is difficult to determine the exact cause of the observed shifts in bacterial communities in response to rotation stage in the wheat-rice cultivation system. Several factors were strongly correlated with plant growth stages, including soil temperature, soil moisture, crop species and growth. Soil temperature varied greatly over the time of this study (from 8.9 to 31.1°C), and this factor was one of the key predictors in the RDA analysis. The effect of temperature could be both direct and indirect. It will have a direct effect on microbial metabolic activity (Schindlbacher et al., 2011) and growth (Bärcenas-Moerno et al., 2009). While numerous indirect effects through crop growth and the decomposition of organic matter (Glanville et al., 2012). The higher 16S rRNA gene copy numbers in May and September corresponding well with the wheat and rice heading stages when the plants had their most vigorous growth (Yoshida, 1981). Soil microbes would be expected to respond differently to fertilizer treatments as plant activity varies through growth stages, and in line with this notion, we observed a significant interactive effect between stage and fertilization treatment on bacterial abundance.

The difference between wheat and rice in the quality and quantity of root exudates could also induce changes in the bacterial community, especially for heterotrophic species (Buyer et al., 2002). Although we did not measure root exudates, previous researchers have reported that root exudates are strongly affected by crop growth stage, and that this relationship in turn influences the succession of bacterial communities (Yang and Crowley, 2000; Duineveld et al., 2001; Xu et al., 2009). In addition, plant species has been acknowledged as an important factor influencing the diversity and structure of microbial communities (Smalla et al., 2001; Kowalchuk et al., 2002), and we also observed difference in soil bacterial communities under wheat versus rice crops, although this impact might be less than pure temporal dynamics.

Flooding of soil between July and September during the rice season had a great influence on the soil bacterial population due to the onset of anoxic conditions (Wang et al., 2015). During this period, oxygen was rapidly depleted in the bulk soil (Liesack et al., 2000), which changed the soil conditions drastically from the wheat ripening (June) to rice tilling (July). These changes would suppress aerobic bacteria while stimulating many previously dormant anaerobic bacteria. Such stimulation was exemplified by the increase in obligate anaerobic bacteria such as *Verrucomicrobia* (Bergmann et al., 2011).

### Effects of Fertilization Regimes Combined with Soil Chemical Properties on Bacterial Communities

The fertilizer treatments significantly impacted the soil chemical properties in our study, thereby affecting the resident bacterial communities. The impacts of different fertilizations on bacterial communities have been reported by previous studies on paddy soil (Yuan et al., 2013; Dong et al., 2014). However, the degree to which such effects persist throughout the whole wheat and rice rotation had not previously been explored, and we observed consistent impact of fertilizer regime despite the backdrop of large temporal changes in microbial community structure.

Concerning the influence of nitrogen fertilizer input, NNF and CF treatments clearly differed, with higher RA of *Cyanobacteria* in the plots that only received phosphorus and potassium fertilizers (NNF). This trend is consistent with the known behavior of *Cyanobacteria* which have the ability to fix nitrogen biologically, and this activity is inhibited by inorganic nitrogen fertilizers inputs (Irisarri et al., 2001; Ramirez et al., 2010; Grandy et al., 2013).

Compared with OIMF treatment, CF improved the soil ammonium and nitrate content and resulted in higher RA of members of the ammonia-oxidizing archaeal *Thaumarchaeota* (Spang et al., 2010; Pester et al., 2011). This group has previously been correlated with soil ammonium contents (Hu et al., 2013), corresponding with our result.

Additions of OFs tend to increase soil organic C and N contents whereas CFs have a much smaller impacts (Lal, 2004). In addition, applications of N as urea, as in the current experiment, lead to acidification of soil (Guo et al., 2010) through the action of ammonium oxidizing bacteria and archaea that produce protons. Hence differences in the microbial populations between organic and inorganic fertilizer treatments are to be expected (Chu et al., 2007; Ge et al., 2008; Zhang et al., 2012). In the present study, higher RAs of *Acidobacteria* but lower RAs of *Proteobacteria* were observed in chemical fertilization treatments as compared to organic fertilization treatments. *Acidobacteria* generally are associated with oligotrophic, slow-growing properties as typical of a K-selected life strategy. Thus, this group is often abundant in soils with low resource availability and low organic C conditions, and it is negatively correlated with soil pH (Fierer et al., 2007). In contrast, *Proteobacteria*, including the α- and β- subclasses, generally exhibit copiotrophic attributes, with higher abundances nutrient-rich high C soils (Fierer et al., 2007; Newton and McMahon, 2011; Zhou et al., 2015). Correlation analyses (**Supplementary Table S3**) supported this ecological distinction, with *Acidobacteria* negatively and α-/β*- Proteobacteria* positively correlated with SOC and TN content. The ratio between *Proteobacteria* and *Acidobacteria* is thought to be an indicator of the nutritional status of the soil ecosystem (Smit et al., 2001; Torsvik and Øvreås, 2002), so it is interesting that this ratio was higher in the treatments receiving pure OF. QPCR also showed that the abundance of *Alphaproteobacteria* and *Betaproteobacteria* classes tended to be higher in the OF treatment. But higher *Acidobacteria* abundance in CF and OIMF treatments, shown in LEfSe, was not detected by the QPCR. This discrepancy with the LEfSe results may be due to the specificity of the QPCR Acid31F primer, which may not amplify all members of the *Acidobacteria* (George et al., 2009; Lee and Cho, 2011). In addition, the low correlation coefficient when comparing QPCR and high throughput sequencing results for the α- and β*- Proteobacteria*, highlights the fact that caution should be exercised when interpreting such PCR-derived results.

It is difficult to know exactly why measures of the bacterial α-diversity were unaffected by fertilization regimes in our study. Clearly, community composition is altered, which alters the RA of different taxa within our experimental study site. Apparently, a range of different selection pressures is at play, i.e., there is not a simple strong selection for a limited number of taxa that thereby lowers diversity.

The grain yields in OF treatment were as low as in the no nitrogen input treatment (**Supplementary Figure S1**) even though OF treatment tended to host more copiotrophic bacterial groups. OF and NNF treatments had a similar extremely low nitrogen availability, thought as the direct factor influencing the crop yield (De Ponti et al., 2012), explaining the low yields in these two treatments. However, one would not expect to identify direct impacts of soil bacterial community size or composition with crop yields. But it is valuable to build up a picture of variations in soil microbial community structure so that, in due course, this can be assessed as a part of sustainability considerations.

It should also be noted that we sampled bulk soil which is inhabited by free-living bacteria such as *Cyanobacteria* and *Proteobacteria* were inhabited (Pii et al., 2015), so the results will be biased toward such organisms rather than those concentrated in the rhizosphere which may show different trends in response to fertilization or plant growth (Ai et al., 2012; Geisseler and Scow, 2014). Thus, future studies comparing rhizosphere and bulk soils will be necessary to gain full understanding of the impacts of fertilizer regime and rotation stage on soil microbial communities in such wheat-rice systems.

## Conclusion

The distinct water managements, climate and crop growth characteristics in wheat and rice rotations dramatically changed the bacterial communities and the RA of specific taxa in our study. The wheat rotation soils tended to have higher aerophilic microbes, while the rice rotation increased the abundance of anaerobic and mesophilic microbes. Furthermore, the application of OF increased the abundance of generally copiotrophic bacterial groups, while application of CF increased the abundance oligotrophic bacteria. Our study provides insights into the successional response of bacterial communities to fertilization and crop growth stages, thereby providing a more complete and robust picture of community responses as compared to the examination of individual time points.

## Author Contributions

JW and YS performed the majority of the experiments. CX and LW gave assistance in data analysis. JW wrote the main manuscript text. QH and QS contributed insightful discussions. All authors reviewed and contributed to the manuscript.

## Conflict of Interest Statement

The authors declare that the research was conducted in the absence of any commercial or financial relationships that could be construed as a potential conflict of interest.

## Supplementary Material

FIGURE S1 **Wheat and rice grain yield for the four treatments (mean ± SE, *n* = 3) in 2013.** The lowercase letters show statistically significantly values among treatments (Tukey, *P*< 0.05). Treatments: NNF, no nitrogen fertilizer; CF, chemical fertilizer; OIMF, organic-inorganic mixed fertilizer; OF, organic fertilizer.

FIGURE S2 **Hierarchical cluster dendrogram based on pairwise Bray–Curtis dissimilarity of bacterial OTU relative abundance**.

TABLE S1 **Soil chemical properties under the four fertilizer regimes during eight growth stages of wheat (March–June) and rice (July–October) rotations**.

Table S2 **Sequence numbers and relative abundances (%) at the phylum level for each sample**.

TABLE S3 **Pearson correlations (r) between abundant phyla and classes (relative abundance > 1%) and soil properties**.
