# Supplementary figures and images for: Wheat and Rice Growth Stages and Fertilization Regimes Alter Soil Bacterial Community Structure, But Not Diversity
Source: Front Microbiol. 2016 Aug 3;7:1207. doi: 10.3389/fmicb.2016.01207 (PMC4971054; doi:10.3389/fmicb.2016.01207)

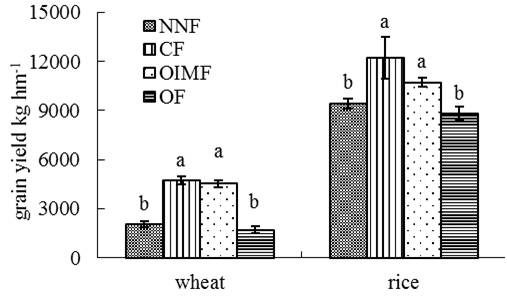

Supplement: FIGURE S1 — Wheat and rice grain yield for the four treatments (mean ± SE, n = 3) in 2013. The lowercase letters show statistically significantly values among treatments (Tukey, P< 0.05). Treatments: NNF, no nitrogen fertilizer; CF, chemical fertilizer; OIMF, organic-inorganic mixed fertilizer; OF, organic fertilizer. [file Image_1.JPEG]

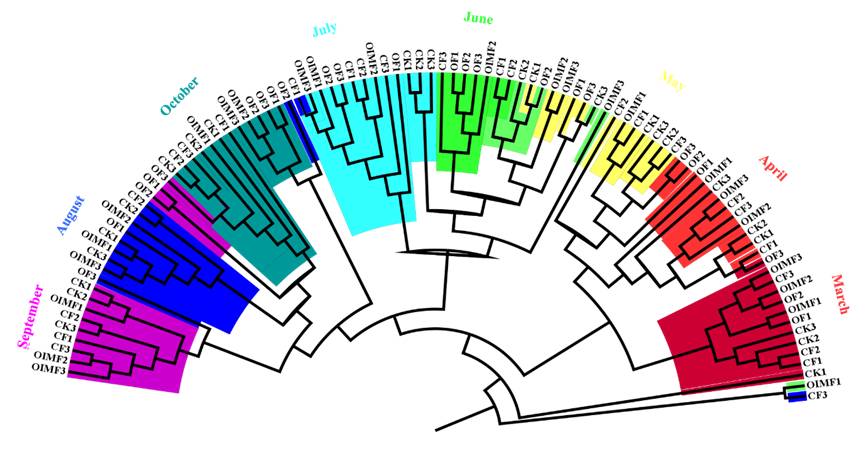

Supplement: FIGURE S2 — Hierarchical cluster dendrogram based on pairwise Bray–Curtis dissimilarity of bacterial OTU relative abundance. [file Image_2.JPEG]
