# Supplementary material for: Wheat and Rice Growth Stages and Fertilization Regimes Alter Soil Bacterial Community Structure, But Not Diversity
Source: Front Microbiol. 2016 Aug 3;7:1207. doi: 10.3389/fmicb.2016.01207 (PMC4971054; doi:10.3389/fmicb.2016.01207)
Supplement: TABLE S1 — Soil chemical properties under the four fertilizer regimes during eight growth stages of wheat (March–June) and rice (July–October) rotations. [file Table_1.DOC]

**Table S1.** Soil chemical properties under the four fertilizer regimes during eight wheat-rice growth stages

| Stage | Fert^a^ | SOC^b^  (g kg^-1^) | TN  (g kg^-1^) | EC  (ms cm^-1^) | pH | NO_3_^-^  (mg kg^-1^) | NH_4_^+^  (mg kg^-1^) | AK  (mg kg^-1^) | AP  (mg kg^-1^) | Soil moisture  (g g^-1^) |
| --- | --- | --- | --- | --- | --- | --- | --- | --- | --- | --- |
| Mar¤ | NNF | 17.79±0.25b | 1.88±0.07a | 0.21±0.003c | 7.43±0.02b | 3.49±0.60b | 0.00±0.00b | 181.12±14.02a | 81.42±30.8a | 0.23±0.01c |
|  | CF | 17.44±0.27b | 1.92±0.14a | 0.26±0.001a | 6.49±0.04d | 52.71±19.53a | 2.35±0.49a | 168.90±2.87a | 86.37±7.46a | 0.29±0.01b |
|  | OIMF | 16.91±0.57b | 1.77±0.21a | 0.21±0.004d | 7.19±0.16c | 8.55±1.09b | 0.13±0.13b | 126.54±6.82b | 58.46±5.52a | 0.29±0.01b |
|  | OF | 20.80±0.82a | 2.08±0.26a | 0.22±0.001b | 7.81±0.01a | 3.10±1.21b | 0.02±0.02b | 140.67±1.63b | 66.02±1.21a | 0.32±0.01a |
| Apr | NNF | 18.57±0.40c | 1.82±0.11a | 0.18±0.001b | 7.59±0.02b | 13.71±0.95b | 1.08±0.28ab | 161.83±2.50a | 65.93±8.13a | 0.27±0.01a |
|  | CF | 18.10±0.28c | 2.09±0.19a | 0.18±0.001b | 6.92±0.03d | 45.75±11.21a | 1.66±0.54a | 107.49±1.82d | 63.51±8.05a | 0.27±0.01a |
|  | OIMF | 19.77±0.17b | 2.05±0.40a | 0.17±0.004c | 7.18±0.02c | 17.58±0.81b | 1.23±0.21ab | 149.40±0.94b | 71.01±9.13a | 0.27±0.02a |
|  | OF | 21.80±0.84a | 2.21±0.24a | 0.24±0.002a | 7.87±0.05a | 11.99±0.87b | 0.72±0.17b | 130.62±3.14c | 67.7±1.19a | 0.29±0.02a |
| May | NNF | 18.48±0.23b | 2.13±0.06b | 0.24±0.001b | 7.50±0.01b | 6.06±0.24c | 0.54±0.37a | 171.25±2.04a | 67.61±3.41a | 0.24±0.01a |
|  | CF | 18.36±0.09b | 2.28±0.03ab | 0.22±0.000c | 6.88±0.03d | 12.33±0.40a | 0.40±0.08a | 102.63±2.35d | 61.28±2.62ab | 0.24±0.02a |
|  | OIMF | 19.62±0.11a | 2.44±0.11a | 0.21±0.001d | 6.97±0.02c | 6.84±0.40b | 0.70±0.10a | 156.8±2.14b | 61.29±3.46ab | 0.24±0.03a |
|  | OF | 20.20±0.56a | 2.26±0.16ab | 0.30±0.002a | 7.90±0.02a | 5.91±0.05c | 0.41±0.05a | 122.77±3.95c | 58.56±5.01b | 0.26±0.01a |
| Jun | NNF | 16.66±0.22d | 2.14±0.01a | 0.18±0.001c | 7.74±0.02b | 5.79±0.03c | 0.28±0.19b | 165.54±1.85a | 68.11±5.95a | 0.26±0.00a |
|  | CF | 17.09±0.06c | 2.16±0.09a | 0.21±0.001b | 7.11±0.04d | 22.62±0.17a | 0.65±0.07b | 115.06±0.71d | 69.78±9.12a | 0.26±0.00a |
|  | OIMF | 18.43±0.29b | 2.63±0.57a | 0.16±0.001d | 7.23±0.03c | 7.34±0.10b | 1.56±0.30a | 134.83±1.31b | 50.73±4.72b | 0.26±0.01a |
|  | OF | 20.70±0.20a | 2.1±0.66a | 0.27±0.000a | 7.90±0.06a | 7.46±0.15b | 0.97±0.66ab | 120.44±1.55c | 71.92±3.01a | 0.26±0.00a |
| Jul | NNF | 16.53±0.21b | 1.99±0.05b | 0.21±0.002b | 7.58±0.01b | 8.76±0.37b | 2.79±0.33b | 179.46±2.66a | 83.96±2.58a | 0.33±0.01a |
|  | CF | 15.55±0.00c | 1.97±0.10b | 0.18±0.001c | 7.64±0.04b | 12.64±1.03a | 6.46±1.87a | 119.19±1.94c | 77.51±0.93b | 0.33±0.01a |
|  | OIMF | 16.16±0.50b | 1.83±0.18b | 0.16±0.001d | 7.58±0.03b | 7.67±1.49b | 3.16±0.38b | 133.15±1.67b | 63.03±1.51d | 0.33±0.01a |
|  | OF | 17.87±0.09a | 2.22±0.04a | 0.25±0.001a | 7.98±0.03a | 2.38±0.48c | 5.55±1.05a | 121.73±2.34c | 68.58±1.59c | 0.32±0.02a |
| Aug | NNF | 15.45±0.41c | 1.92±0.06b | 0.22±0.023b | 7.75±0.05b | 2.10±0.28a | 2.36±0.30a | 137.56±2.85a | 63.13±3.17c | 0.30±0.01b |
|  | CF | 16.32±0.22d | 1.92±0.04b | 0.20±0.000b | 7.75±0.06b | 11.70±1.32c | 5.81±0.13b | 90.94±1.26d | 65.31±2.06bc | 0.30±0.02b |
|  | OIMF | 17.43±0.10b | 2.02±0.07ab | 0.20±0.001b | 7.65±0.03c | 6.96±1.63b | 4.98±2.66ab | 128.70±0.56b | 71.11±4.55b | 0.32±0.01ab |
|  | OF | 20.26±0.60a | 2.19±0.17a | 0.32±0.003a | 7.95±0.03a | 7.35±3.64b | 2.35±0.05b | 120.36±0.55c | 79.49±2.86a | 0.33±0.01a |
| Sep | NNF | 18.86±1.70ab | 2.14±0.16a | 0.20±0.000b | 7.55±0.03b | 1.23±0.33b | 4.54±1.31a | 163.11±1.93a | 71.84±9.99b | 0.31±0.01a |
|  | CF | 19.19±0.43ab | 2.08±0.36a | 0.17±0.001d | 7.36±0.01c | 4.44±0.08a | 3.66±0.55a | 109.13±2.15d | 75.5±2.08b | 0.32±0.01a |
|  | OIMF | 18.42±0.35b | 2.18±0.17a | 0.18±0.001c | 7.32±0.04c | 0.77±0.20c | 6.05±4.15a | 134.29±0.87b | 73.88±10.7b | 0.32±0.01a |
|  | OF | 20.67±0.86a | 2.30±0.28a | 0.31±0.006a | 7.88±0.07a | 0.49±0.25c | 3.15±0.10a | 118.79±1.46c | 90.01±7.18a | 0.33±0.01a |
| Oct | NNF | 17.54±0.26b | 1.99±0.27b | 0.20±0.002c | 7.51±0.03b | 7.00±2.17a | 4.35±1.41a | 153.58±0.79a | 57.04±5.32b | 0.26±0.01b |
|  | CF | 18.36±0.62b | 2.51±0.08a | 0.22±0.000b | 7.05±0.03c | 9.07±0.64a | 5.17±0.67a | 110.68±1.82d | 73.43±2.78a | 0.25±0.01b |
|  | OIMF | 18.00±0.81b | 2.36±0.10a | 0.20±0.003d | 7.08±0.02c | 7.17±0.78a | 5.66±0.73a | 147.06±3.23b | 64.49±5.54b | 0.27±0.02b |
|  | OF | 21.96±0.23a | 2.47±0.12a | 0.35±0.002a | 7.78±0.06a | 6.99±3.05a | 4.95±1.02a | 128.68±3.06c | 74.04±4.73a | 0.30±0.01a |

^a^ Fert stands for four fertilizer regimes: NNF no nitrogen fertilizer, CF chemical fertilizer, OIMF organic-inorganic mixed fertilizer, OF organic fertilizer.

^b^ Values are presented as mean±SE (n=3), different letters of the same sampling stage indicate significant differences among fertilizer treatments by one-way ANOVAs (Tukey, *P* < 0.05), SOC soil organic carbon, TN total nitrogen, EC electrical conductivity, AK available K, AP available P.

¤ Data of SOC, TN, NO_3_^-^ and NH_4_^+^ across Mar to Jun derived from Wang et al. (2016).
