# Supplementary material for: Wheat and Rice Growth Stages and Fertilization Regimes Alter Soil Bacterial Community Structure, But Not Diversity
Source: Front Microbiol. 2016 Aug 3;7:1207. doi: 10.3389/fmicb.2016.01207 (PMC4971054; doi:10.3389/fmicb.2016.01207)
Supplement: Table S2 — Sequence numbers and relative abundances (%) at the phylum level for each sample. [file Table_2.DOCX]

**Table S2.** Sequence numbers and relative abundances (%) at the phylum level for each sample

| Stage | Fert^a^ | Proteo^b^ | Acido | Actino | Thaum | Bacteroi | Chloro | Firmi | Nitro | Planc | Verru | Sequences |
| --- | --- | --- | --- | --- | --- | --- | --- | --- | --- | --- | --- | --- |
| Mar | NNF | 34.2±0.8a | 8.8±0.7a | 10.3±1.3a | 1.6±0.7a | 1.5±0.6a | 6.4±2.6a | 1.5±0.4a | 0.8±0.1a | 2.0±0.5a | 1.7±0.2a | 13131±3517a |
|  | CF | 32.4±2.3a | 8.5±0.6a | 9.7±0.4a | 1.2±0.4a | 1.0±0.1a | 7.5±0.3a | 1.3±0.1a | 0.9±0a | 1.6±0.1a | 1.9±0.1a | 13607±220a |
|  | OIMF | 33.9±1.7a | 9.0±0.1a | 10.1±0.4a | 0.8±0.1a | 0.8±0.1a | 6.8±0.6a | 1.2±0a | 1.0±0.1a | 1.5±0.1a | 1.8±0a | 13815±3526a |
|  | OF | 34.5±0.9a | 8.5±0.3a | 9.5±1.1a | 1.6±0.4a | 1.5±0.6a | 7.0±0.6a | 1.4±0.2a | 0.9±0.1a | 1.6±0.2a | 1.9±0.2a | 20219±7183a |
| Apr | NNF | 34.4±1.3ab | 8.7±0.2a | 6.5±0.5a | 1.9±0.3ab | 4.1±0.2a | 7.4±0.8a | 1.4±0.1a | 1.0±0.1a | 1.6±0.2a | 2.4±0.1a | 13633±1880a |
|  | CF | 33.0±1.4b | 8.3±0.3a | 6.5±0.1a | 2.3±0.5a | 2.6±0.3bc | 7.4±0.3a | 1.4±0.1a | 1.1±0.1a | 1.4±0.1ab | 2.3±0.1a | 17606±3053a |
|  | OIMF | 37.0±1.4a | 8.6±0.1a | 6.9±0.1a | 1.2±0.2bc | 3.0±0.2b | 6.8±0.7a | 1.4±0a | 1.0±0.1a | 1.2±0b | 2.3±0.1a | 12571±4950a |
|  | OF | 36.6±1.2a | 8.4±0.2a | 6.6±0.2a | 1.0±0.2c | 2.1±0.2c | 7.1±0.8a | 1.5±0.2a | 1.0±0a | 1.2±0.1b | 2.2±0.1a | 12841±3752a |
| May | NNF | 35.0±1.3a | 9.3±0.1a | 6.5±0.2a | 1.0±0.1b | 2.0±0.1a | 6.5±0a | 1.4±0.1a | 0.9±0.1a | 1.3±0.1a | 2.4±0a | 11510±2167a |
|  | CF | 35.9±0.5a | 8.7±0.2a | 6.1±0.2a | 1.8±0.5a | 2.3±0.2a | 7.2±0.4a | 1.4±0a | 1.0±0.1a | 1.3±0.1a | 2.5±0.1a | 14032±2825a |
|  | OIMF | 36.9±3.4a | 8.7±0.3a | 6.3±0.5a | 1.2±0.2ab | 1.8±0.3a | 6.5±1.4a | 1.3±0.1a | 1.1±0.1a | 1.2±0.4a | 2.1±0.4ab | 12021±3640a |
|  | OF | 36.6±0.7a | 8.5±0.5a | 6.6±0.1a | 1.3±0ab | 1.8±0.1a | 7.2±0.5a | 1.3±0a | 1.0±0a | 1.3±0.1a | 1.7±0b | 12075±1076a |
| Jun | NNF | 34.4±1.0a | 8.9±0.2a | 7.2±0.3a | 1.1±0 | 1.3±0.1c | 6.5±0.2a | 1.3±0a | 1.0±0a | 1.2±0.1a | 1.8±0.2a | 14033±2987a |
|  | CF | 35.7±0.5a | 8.7±0.1a | 6.2±0.3a | 1.5±0.3 | 1.5±0bc | 7.7±0.5a | 1.2±0.1a | 1.1±0.1a | 1.3±0a | 2.1±0.2a | 19920±10973a |
|  | OIMF | 42.1±11.1a | 9.0±0.3a | 6.3±0.9a | 0.9±0.1 | 1.8±0.2ab | 4.9±2.4a | 1.5±0.4a | 0.9±0.4a | 1.1±0.3a | 2.1±0.7a | 10431±848a |
|  | OF | 36.2±1.3a | 8.7±0.4a | 6.1±0.2a | 1.1±0.1 | 1.9±0a | 5.8±0.7a | 1.3±0a | 1.0±0a | 1.3±0.1a | 2.3±0.1a | 12058±951a |
| Jul | NNF | 33.0±1.4ab | 9.3±0.5a | 5.3±0.2a | 1.3±0.5 | 1.7±0.1a | 6.8±0.2a | 1.2±0.1b | 1.0±0.1c | 1.4±0.2a | 2.7±0b | 14679±3162a |
|  | CF | 32.2±0.2b | 9.8±0.1a | 4.4±0.1b | 1.4±0.4 | 1.4±0.2b | 7.1±0.5a | 1.4±0.1a | 1.2±0b | 1.1±0b | 3.3±0a | 13740±5449a |
|  | OIMF | 34.8±0.4a | 10.1±0.5a | 4.5±0.1b | 1.0±0 | 1.3±0b | 6.7±0.1a | 1.3±0ab | 1.3±0a | 1.0±0b | 3.1±0.3ab | 14358±3426a |
|  | OF | 32.5±0.2b | 9.4±0.1a | 4.4±0.2b | 1.0±0.1 | 1.5±0.1ab | 6.7±0.2a | 1.5±0.1a | 1.2±0b | 1.1±0.1b | 2.9±0.3ab | 14135±774a |
| Aug | NNF | 35.9±9.5a | 8.7±0.2a | 4.1±0a | 2.0±0.2 | 1.6±0.1a | 7.3±0.2a | 1.5±0a | 1.1±0a | 1.4±0.1a | 2.9±0.1a | 12688±816a |
|  | CF | 31.3±0.3a | 10.9±3.6a | 6.0±2.9a | 1.5±1.5 | 1.3±0.1a | 6.4±3.6a | 1.7±0.5a | 0.9±0.5a | 1.3±0.2a | 2.4±0.7a | 16335±14113a |
|  | OIMF | 31.0±0.4a | 8.6±0.1a | 4.2±0.1a | 1.9±0.2 | 1.6±0.1a | 7.4±0.5a | 1.4±0a | 1.2±0a | 1.3±0a | 2.8±0a | 12033±3305a |
|  | OF | 32.5±0.7a | 8.3±0.2a | 4.2±0.3a | 1.8±1.0 | 1.8±0.6a | 7.1±1.0a | 1.4±0.1a | 1.1±0.1a | 1.5±0.2a | 2.8±0.4a | 14537±5648a |
| Sep | NNF | 30.3±0.5a | 9.2±0.3a | 4.3±0.1a | 2.1±0.2 | 1.9±0b | 8.9±0.6a | 1.5±0a | 1.1±0.1ab | 1.6±0.1a | 3.2±0.1ab | 10559±4472a |
|  | CF | 30.0±0.2a | 8.8±0.1ab | 3.8±0.1b | 2.2±0.4 | 2.1±0.1a | 9.8±0.5a | 1.5±0.1a | 1.2±0a | 1.3±0a | 3.5±0.1a | 15420±557a |
|  | OIMF | 29.9±1.2a | 9.3±0.2a | 3.9±0.2ab | 2.4±0.5 | 2.2±0.1a | 9.1±0.4a | 1.6±0.1a | 1.2±0.1a | 1.5±0.1a | 3.3±0ab | 13315±2871a |
|  | OF | 32.0±1.4a | 8.4±0.3b | 4.3±0.2a | 2.2±0.3 | 1.5±0c | 9.2±0.9a | 1.5±0a | 1.0±0.1b | 1.3±0.1a | 3.0±0.2b | 14365±3978a |
| Oct | NNF | 32.5±1.9a | 8.9±0.3a | 4.2±0.2a | 1.7±0.2 | 1.6±0.1b | 8.8±0.3a | 1.4±0ab | 1.1±0ab | 1.2±0.1a | 3.4±0.1a | 13665±1866a |
|  | CF | 31.8±0.3a | 8.9±0.3a | 4.6±0.3a | 1.7±0.4 | 1.5±0.1ab | 8.7±0.9a | 1.5±0.1a | 1.0±0b | 1.3±0a | 3.1±0.1ab | 13023±5043a |
|  | OIMF | 34.3±2.6a | 8.0±0.5ab | 4.8±0.3a | 1.5±0.3 | 2.1±0.3a | 7.6±0.7a | 1.3±0.1b | 1.1±0.1a | 1.1±0.2a | 3.0±0.3ab | 14414±2915a |
|  | OF | 35.3±1.6a | 7.3±0.3b | 4.6±0.3a | 2.0±0.6 | 2.1±0.1a | 8.3±0.8a | 1.3±0b | 1.0±0b | 1.3±0.2a | 2.8±0b | 12681±2926a |

^a^ Fert indicates the four fertilizer regimes: NNF no nitrogen fertilizer, CF chemical fertilizer, OIMF organic-inorganic mixed fertilizer, OF organic fertilizer.

^b^ Phyla with the relative abundances higher than 1 % are shown. Values are presented as mean±SD (n=3), different letters of the same sampling stage indicate significant differences among fertilizer treatments by one-way ANOVAs (Tukey, *P* < 0.05). Phylum abbreviations: Proteo, *Proteobacteria* Acido, *Acidobacteria*; Actino, *Actinobacteria*; Thaum, *Thaumarchaeota*; Bacteroi, *Bacteroidetes*; Chloro, *Chloroflexi*; Firmi, *Firmicutes*; Nitro, *Nitrospirae*; Planc, *Planctomycetes*; Verru, *Verrucomicrobia*.
