# Supplementary material for: Wheat and Rice Growth Stages and Fertilization Regimes Alter Soil Bacterial Community Structure, But Not Diversity
Source: Front Microbiol. 2016 Aug 3;7:1207. doi: 10.3389/fmicb.2016.01207 (PMC4971054; doi:10.3389/fmicb.2016.01207)
Supplement: TABLE S3 — Pearson correlations (r) between abundant phyla and classes (relative abundance > 1%) and soil properties. [file Table_3.DOCX]

**Table S3** Pearson correlations (r) between abundant phyla and classes (relative abundance>1%) and soil properties.

|  | SOC^a^ | TN | EC | pH | NO_3_^-^ | NH_4_^+^ | AK | AP | Soil moisture | temperature |
| --- | --- | --- | --- | --- | --- | --- | --- | --- | --- | --- |
| *Proteobacteria* | 0.214* | 0.387** | 0.056 | -0.077 | 0.126 | -0.295** | -0.024 | -0.271** | -0.337** | -0.205* |
| *Actinobacteria* | 0.039 | -0.197 | -0.020 | -0.294** | 0.327** | -0.609** | 0.331** | 0.002 | -0.375** | -0.725** |
| *Acidobacteria* | -0.455** | -0.228* | -0.343** | 0.069 | -0.106 | 0.200 | -0.055 | -0.017 | 0.179 | 0.267** |
| *Chloroflexi* | 0.087 | -0.067 | 0.070 | -0.053 | -0.059 | 0.335** | -0.143 | 0.081 | 0.225* | 0.069 |
| *Verrucomicrobia* | -0.122 | -0.002 | -0.099 | 0.148 | -0.292** | 0.694** | -0.213* | 0.125 | 0.464** | 0.568** |
| *Planctomycetes* | 0.031 | -0.355** | 0.109 | -0.087 | 0.093 | -0.240* | 0.318** | 0.335** | -0.086 | -0.367** |
| *Firmicutes* | 0.067 | 0.142 | -0.099 | 0.116 | -0.166 | 0.186 | -0.130 | 0.218* | 0.061 | 0.127 |
| *Bacteroidetes* | 0.336** | 0.058 | -0.138 | -0.058 | 0.094 | -0.126 | 0.084 | -0.030 | -0.216* | -0.316** |
| *Gemmatimonadetes* | -0.200 | -0.200 | -0.280** | -0.278** | 0.221* | -0.519** | 0.239* | 0.011 | -0.422** | -0.391** |
| *Nitrospirae* | -0.199 | -0.131 | -0.182 | 0.121 | -0.138 | 0.365** | -0.178 | -0.078 | 0.378** | 0.435** |
| *Thaumarchaeota* | 0.076 | 0.043 | 0.046 | -0.004 | -0.022 | 0.261* | -0.161 | 0.297** | 0.195 | 0.124 |
| *Betaproteobacteria* | 0.300^**^ | 0.12 | 0.380^**^ | 0.135 | 0.032 | -0.276^**^ | -0.062 | -0.183 | -0.217^*^ | -0.083 |
| *Alphaproteobacteria* | 0.258^*^ | 0.176 | -0.037 | -0.232^*^ | 0.283^**^ | -0.563^**^ | 0.18 | -0.192 | -0.475^**^ | -0.598^**^ |
| *Deltaproteobacteria* | 0.021 | 0.393^**^ | -0.079 | -0.047 | -0.135 | .358^**^ | -0.128 | -0.081 | 0.187 | 0.239^*^ |
| *Gammaproteobacteria* | 0.008 | 0.258^*^ | -0.13 | -0.107 | 0.097 | -0.14 | -0.046 | -0.144 | -0.215^*^ | -0.1 |
| *Anaerolineae* | 0.074 | -0.07 | 0.034 | -0.049 | -0.087 | 0.393^**^ | -0.132 | 0.107 | 0.299^**^ | 0.109 |
| *Acidobacteria_Gp6* | -0.181 | -0.146 | -0.071 | 0.066 | 0.025 | -0.144 | 0.028 | -0.007 | -0.163 | -0.001 |
| *Subdivision3* | -0.104 | 0 | -0.052 | 0.169 | -0.290^**^ | 0.676^**^ | -0.206^*^ | 0.126 | 0.465^**^ | 0.576^**^ |
| *Acidobacteria_Gp3* | -0.296^**^ | -0.131 | -0.277^**^ | 0.053 | -0.245^*^ | 0.591^**^ | -0.163 | 0.113 | 0.547^**^ | 0.489^**^ |
| *Planctomycetia* | 0.032 | -0.354^**^ | 0.111 | -0.085 | 0.092 | -0.241^*^ | 0.320^**^ | 0.336^**^ | -0.086 | -0.367^**^ |
| *Acidobacteria_Gp16* | -0.219^*^ | -0.393^**^ | -0.159 | -0.355^**^ | 0.360^**^ | -0.403^**^ | 0.209^*^ | -0.027 | -0.164 | -0.579^**^ |
| *Acidobacteria_Gp17* | -0.043 | -0.121 | 0.016 | -0.105 | 0.286^**^ | -0.389^**^ | 0.115 | -0.274^**^ | -0.157 | -0.417^**^ |
| *Nitrospira* | -0.199 | -0.131 | -0.182 | 0.121 | -0.138 | 0.365^**^ | -0.178 | -0.078 | 0.378^**^ | 0.435^**^ |

^a^ Abbreviations: SOC stands for soil organic carbon, TN stands for total nitrogen, EC stands for electrical conductivity, AK stands for available K, AP stands for available P.

*P<0.05; **P<0.01
